# Metformin Attenuates Postinfarction Myocardial Fibrosis and Inflammation in Mice

**DOI:** 10.3390/ijms22179393

**Published:** 2021-08-30

**Authors:** Halyna Loi, Solomiia Kramar, Charlotte Laborde, Dimitri Marsal, Nathalie Pizzinat, Daniel Cussac, Jerome Roncalli, Frederic Boal, Helene Tronchere, Oleksandra Oleshchuk, Mykhaylo Korda, Oksana Kunduzova

**Affiliations:** 1 Department of Pharmacology and Clinical Pharmcology, I. Horbachevsky Ternopil National Medical University, 46009 Ternopil, Ukraine; loy@tdmu.edu.ua (H.L.); kramar.solomija@gmail.com (S.K.); lesyaoleh@gmail.com (O.O.); Mykhaylo.Korda@tdmu.edu.ua (M.K.); 2 National Institute of Health and Medical Research (INSERM) U1297, CEDEX 4, 31432 Toulouse, France; charlotte.laborde@inserm.fr (C.L.); Dimitri.Marsal@inserm.fr (D.M.); Nathalie.Pizzinat@inserm.fr (N.P.); Daniel.Cussac@inserm.fr (D.C.); Jerome.Roncalli@inserm.fr (J.R.); Frederic.Boal@inserm.fr (F.B.); Helene.Tronchere@inserm.fr (H.T.); 3 University of Toulouse III, CEDEX 9, 31062 Toulouse, France

**Keywords:** metformin, myocardial infarction, fibrosis, inflammation, cardiac remodeling

## Abstract

Diabetes is a major risk factor for the development of cardiovascular disease with a higher incidence of myocardial infarction. This study explores the role of metformin, a first-line antihyperglycemic agent, in postinfarction fibrotic and inflammatory remodeling in mice. Three-month-old C57BI/6J mice were submitted to 30 min cardiac ischemia followed by reperfusion for 14 days. Intraperitoneal treatment with metformin (5 mg/kg) was initiated 15 min after the onset of reperfusion and maintained for 14 days. Real-time PCR was used to determine the levels of COL3A1, αSMA, CD68, TNF-α and IL-6. Increased collagen deposition and infiltration of macrophages in heart tissues are associated with upregulation of the inflammation-associated genes in mice after 14 days of reperfusion. Metformin treatment markedly reduced postinfarction fibrotic remodeling and CD68-positive cell population in mice. Moreover, metformin resulted in reduced expression of COL3A1, αSMA and CD68 after 14 days of reperfusion. Taken together, these results open new perspectives for the use of metformin as a drug that counteracts adverse myocardial fibroticand inflammatory remodeling after MI.

## 1. Introduction

Diabetes mellitus is a major risk factor for the incidence of myocardial infarction (MI) and cardiovascular morbidity and mortality [1]. Patients with diabetes have over twice the risk of developing heart failure (HF) than patients without diabetes mellitus [2]. Experimental and clinical investigations suggest that the diabetic heart is more sensitive to ischemic injury than the nondiabetic heart [3]. Myocardial ischemia/reperfusion (I/R) injury is a complex pathophysiological event, resulting in serious acute and chronic myocardial damage linked to adverse myocardial remodeling processes [4,5,6]. Cardiac remodeling is defined as a group of molecular, cellular and interstitial changes that contribute to the progression of heart disease [7]. Patients with diabetes frequently have echocardiographic evidence of adverse myocardial remodeling; both increased left ventricular mass and dilatation have been reported [8], and these phenotypes are well-known predictors of HF.

Cardiac fibrosis and inflammation are central to the pathogenesis of cardiovascular and metabolic disorders. Activated fibroblasts and immune cells are critically involved in the resolution of aberrant remodeling processes leading to the decline in cardiac function [9]. The inflammatory components vary across different fibrotic conditions but share macrophage-mediated responses, with the abundant release of profibrotic factors as a common feature after cardiac injury [10,11]. The inflammatory component is primarily composed of time-dependent infiltration of neutrophils and macrophages in the ischemic heart [12]. Several studies have shown significant elevation of CD68 expression in macrophages in response to inflammatory stimuli [13]. Inflammation-associated cytokines including tumor necrosis factor-alpha (TNF-α) and interleukin6 (IL-6) trigger additional cellular inflammatory cascades and activate matrix metalloproteinases that contribute to the development of fibrotic reprogramming of the heart and dysfunction [14,15]. In fibrotic tissue remodeling, activated cardiac fibroblasts change their phenotype and differentiate into myofibroblasts, characterized by expression of α-smooth muscle actin (αSMA) and production of extracellular matrix (ECM) proteins [16,17,18,19]. Cardiac myofibroblasts contribute to the structural and functional changes in the heart by increased deposition of ECM components, predominantly collagen types I and III, within the interstitium by regulating autocrine/paracrine factors [20,21].

Metformin, a first-line pharmacological agent for type 2 diabetes mellitus (T2D) [22] has been reported to reduce major cardiovascular events and death [23]. Metformin preserves cardiac function and prevents the incidence of MI in patients with diabetes [23]. Compared with other glucose-lowering agents, the use of metformin is associated with a reduced risk of cardiovascular mortality and morbidity in patients with T2D [24]. Both the link between diabetes and HF and the molecular mechanism of action of metformin remain incompletely understood. Inflammation and fibrosis are the final common pathological manifestations in diabetes and HF [25], and interventions aimed at reducing fibrotic and inflammatory remodeling are of paramount importance.

In the present study, we demonstrate that posttreatment with metformin counteracts fibrotic and inflammatory remodeling in a mouse model of cardiac I/R injury.

## 2. Results

### 2.1. Metformin Treatment Decreases Fibrotic Remodeling in a Mouse Model of Cardiac I/R Injury

**Figure 1:**
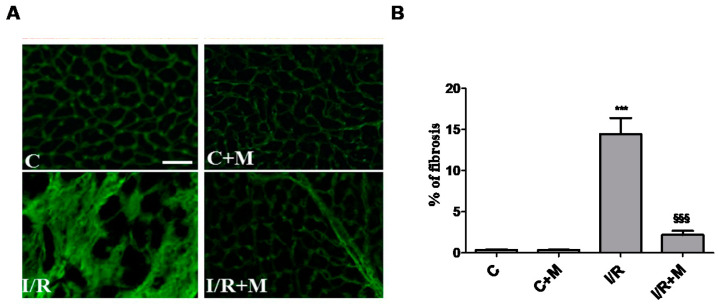
Metformin treatment prevents fibrotic remodeling in postinfarcted myocardium. (**A**) Representative images of wheat germ agglutinin (WGA) staining of frozen heart tissue sections; scale bar is 25 µm. Frozen sections from vehicle- or metformin-treated mice subjected to 30 min of cardiac ischemia and 14 days of reperfusion were stained with WGA. Metformin treatment (5 mg/kg, i.p.) started 15 min after the onset of reperfusion and continued for 14 days. (**B**) Quantification of fibrotic area from (**A**), *n* = 3–5. Data are presented as mean ± SEM. One-way ANOVA followed by Bonferroni’s post hoc test: *** *p* < 0.001 vs. control (C), §§§ *p* < 0.001 vs. I/R.

To examine the translational potential of metformin, an in vivo study was designed to determine whether treatment with metformin (5 mg/kg i.p.) initiated 15 min after the onset of reperfusion and maintained for 14 days counteracts I/R-induced fibrotic and inflammatory responses in mice. Post-MI cryosections of the hearts were stained by both wheatgerm agglutinin (WGA) and Sirius Red. Quantification of fibrotic tissue in cardiac sections using fluorescent probe WGA (Figure 1A,B) revealed that I/R increased collagen accumulation in the myocardium (14.43 ± 1.95% in I/R group versus 0.35 ± 0.05% in С group, *p* < 0.001), while metformin treatment prevented fibrotic remodeling in mice subjected to I/R (2.20 ± 0.48% in I/R + М group versus 14.43 ± 1.95% in I/R group, *p* < 0.001).

**Figure 2:**
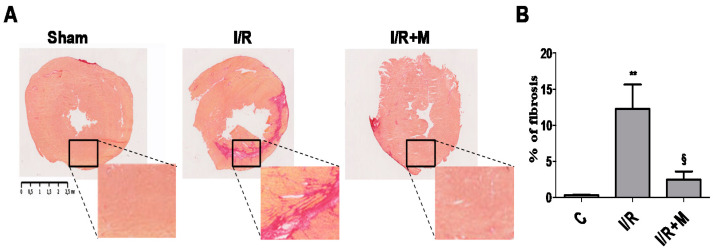
Metformin attenuates cardiac fibrosis in postinfarcted myocardium. Metformin treatment prevents fibrotic remodeling in postinfarcted myocardium. (**A**) Representative images of Sirius Red staining of frozen heart tissue sections from vehicle- or metformin-treated mice subjected to 30 min of cardiac ischemia and 14 days of reperfusion. Metformin treatment (5 mg/kg, i.p.) started 15 min after the onset of reperfusion and continued for 14 days. (**B**) Quantification of fibrotic area from (**A**), *n* = 4–5. Data are presented as mean ± SEM. One-way ANOVA followed by Bonferroni’s post hoc test: ** *p* < 0.01 vs. control (C), § *p* < 0.001 vs. I/R.

Histological analyses of cardiac sections stained with Sirius Red (Figure 2A,B) demonstrated that I/R triggered myocardial fibrosis (12.26 ± 3.36% in I/R group versus 0.33 ± 0.07% in control (С) group, *p* < 0.01). Treatment of mice with metformin was able to attenuate I/R-induced collagen deposition in the myocardium (2.49 ± 1.13% in I/R + М group versus 12.26 ± 3.36% in I/R group, *p* < 0.05).

**Figure 3:**
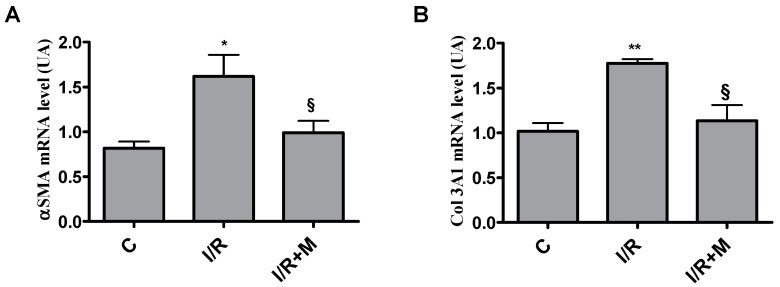
Metformin treatment downregulates profibrotic genes in postinfarcted myocardium. (**A**) qRT-PCR quantification of the expression level of the myofibroblast marker αSMA. (**B**) qRT-PCR quantification of the expression level of the collagen type III. Data are presented as mean ± SEM. One-way ANOVA followed by Bonferroni’s post hoc test: * *p* < 0.05 vs. control (C), ** *p* < 0.01 vs. control (C), § *p* < 0.05 vs. I/R.

Increased collagen deposition in I/R group is associated with a significant increase in Col3 and α-SMA mRNA expression as compared to the control group. However, metformin treatment reduced I/R-induced Col3 and αSMA expression levels (Figure 3A,B).

### 2.2. Metformin Exerts Anti-Inflammatory Activity in Cardiac Section

**Figure 4:**
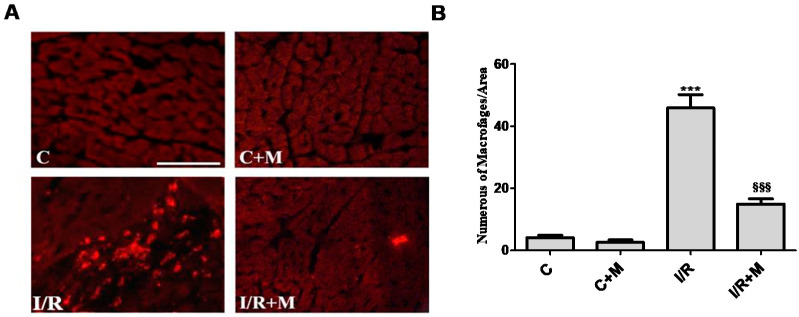
Metformin exerts anti-inflammatory activity in postinfarcted myocardium. (**A**) CD68 immunostaining was performed in order to detect inflammatory cell populations in cardiac sections from vehicle- or metformin-treated mice subjected to 30 min of cardiac ischemia and 14 days of reperfusion. Metformin treatment (5 mg/kg, i.p.) started at 15 min of reperfusion and continued for 14 days. Scale bar is 100 µm. (**B**) Quantification of inflammatory cells from (**A**), *n* = 4–5. One-way ANOVA followed by Bonferroni’s post hoc test: *** *p* < 0.001 vs. control (C), §§§ *p* < 0.001 vs. I/R.

We next explored the role of metformin in inflammatory processes after cardiac I/R injury. Analysis of CD68 immunostaining revealed a significant increase in the number of inflammatory CD68-positive cells (Figure 4A,B) in I/R mice (38.80 ± 4.30%) as compared to control mice (2.4 ± 0.75%), *p* < 0.001. Our data demonstrated a reduction in inflammatory cell population in cardiac section after I/R from metformin-treated mice (14.40 ± 2.32% in I/R + М group versus 38.80 ± 4.30% in I/R group, *p* < 0.001).

**Figure 5:**
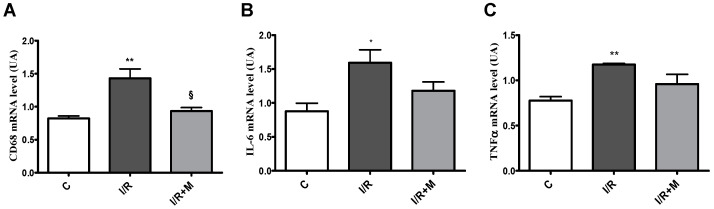
Effect of metformin treatment on cardiac expression of CD68, IL-6 and TNF-αin a mouse model of I/R injury. qRT-PCR quantification of the expression level of CD68 (**A**), IL-6 (**B**) and TNF-α (**C**), *n* = 4–5. Data are presented as mean ± SEM. One-way ANOVA followed by Bonferroni’s post hoc test: * *p* < 0.05 vs. control (C), ** *p* < 0.01 vs. control (C), § *p* < 0.05 vs. I/R.

As shown in Figure 5A, cardiac I/R induced CD68 mRNA expression in cardiac tissue as compared to control mice. However, treatment with metformin prevented an increase in CD68 mRNA level in cardiac sections after I/R. In addition, cardiac expression of key proinflammatory genes, including TNF-α and IL-6, was evaluated in cardiac sections from vehicle- or metformin-treated mice after 14 days of I/R (Figure 5B,C). As shown in Figure 5B,C, we found that I/R promoted upregulation of TNF-α and IL-6. However, we did not detect significant differences inTNF-α and IL-6 mRNA expression levels after 14 days of I/R.

**Figure 6:**
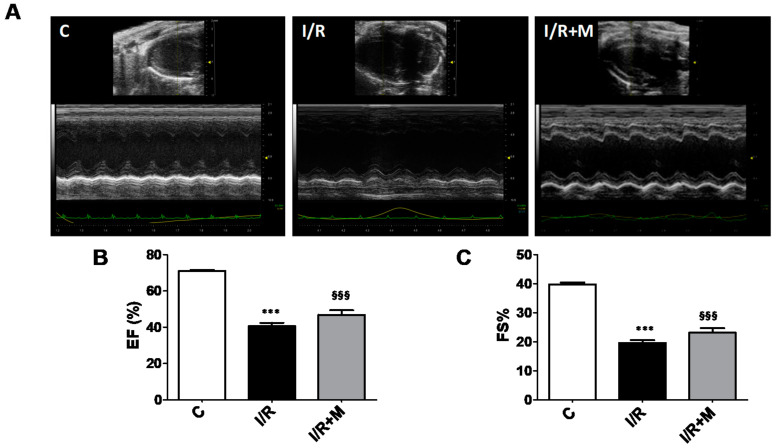
Echocardiographic functional parameters of mice after 14 days of cardiac I/R. (**A**) Representative 2D-M-Mode echocardiographic images of vehicle- or metformin-treated mice subjected to 14 days of I/R. (**B**) Ejection fraction (EF) and (**C**) fractional shortening (FS) invehicle- or metformin-treated I/R mice (*n* = 3 per group). Data are presented as mean ± SEM. One-way ANOVA followed by Bonferroni’s post hoc test: *** *p* < 0.001, §§§ *p* < 0.001 vs. control (C).

We next compared the cardiac function in vehicle- and metformin-treated mice subjected to 14 days of I/R (Figure 6). As shown in Figure 6A–C, echocardiographic assessment of cardiac function showed a decline in ejection fraction (EF) and fraction shortening (FS) in I/R-challenged mice as compared to control. In metformin-treated mice, both EF and FS tended to increase following I/R injury, but this did not reach significance (Figure 6B,C). The echocardiographic parameters are shown in Supplementary Table S1.

## 3. Discussion

Metformin, the most widely prescribed drug therapy for T2D, has pleiotropic benefits, in addition to its capacity to lower elevated blood glucose levels [26]. Nonetheless, uncertainty still exists with regard to its effects on reactive cardiac remodeling linked to HF. In the present study, we show that low-dose metformin counteracts abnormal myocardial fibrosis in postinfarcted hearts in mice. In addition, we demonstrate that metformin blunts inflammatory response in injured myocardium by decreasing macrophage accumulation. These findings indicate that metformin treatment attenuates myocardial cardiac fibrotic remodeling associated with inflammation in the early postinfarction period.

In response to the loss of cardiac cells following MI, the cardiac interstitium is dynamically remodeled and contributes to the pathogenesis of HF. In acute MI, ischemia leads to initial cell death, followed by an inflammatory response with replacement fibrosis in the infarct region in the myocardium [21]. The cellular changes prime both the infarcted and noninfarcted areas for progressive ventricular dysfunction that eventually can lead to HF [4,7]. Accumulating evidence indicates that altering the initial cellular responses to I/R may enhance cardiomyocyte survival and ultimately preserve cardiac function following MI [27]. We have recently demonstrated that metformin prevents apoptotic cell death in both in vitro and in vivo models of myocardial I/ R injury, suggesting a role of metformin in cardiomyocyte death. Furthermore, we have shown that metformin protects against postinfarct cardiac hypertrophy and regulates myocardial expression of brain-like natriuretic peptide (BNP) [28]. It is well known that pathological hypertrophy is closely associated with apoptotic and necrotic cell death and the loss of myocytes is replaced with excessive collagen accumulation in the myocardium, resulting in interstitial fibrosis [29]. Although the initial activation of fibrotic machinery is crucial for preventing rupture of the ventricular wall, an exaggerated fibrotic response and reactive fibrosis outside the injured area are detrimental as they lead to progressive impairment of cardiac function and eventually to heart disease [30]. In this study, we found that metformin blunts pathological fibrotic remodeling in mice subjected to 14 days of cardiac I/R. These findings are in line with previous evidence from an animal model of HF suggesting that metformin attenuates myocardial fibrosis [31,32]. The fundamental mechanism underlying the excessive fibrotic remodeling after I/R injury is largely obscure but has been the subject of intense research. The majority of studies assign a key role in fibrotic progression to TGF-β, which executes its biological function by downstream activation of the Smad signaling pathway [31]. TGF-β1, the most abundant isoform of TGF-β family members, can be secreted by cardiac cells and infiltrated inflammatory cells [33]. Inhibition of TGF-β1 or its downstream signaling pathways substantially limits tissue fibrosis in a wide range of disease models, whereas overexpression of TGF-β1 induces fibrotic remodeling [33]. In the context of myocardial fibrosis, Wang et al. reported that metformin can counteract the TGF-β_1_–Smad3 signaling pathway including TGF-β_1_ production, phosphorylation of Smad3 and nuclear translocation of Smad3 [31]. Metformin might also activate AMPK, which is expressed in cardiac tissue. Several studies have demonstrated that metformin protects the heart against myocardial I/R injury by suppressing oxidative stress and inflammation through AMPK activation [34,35,36]. However, the AMPK inhibitor (compound C) and adenovirus transduction with AMPKα1-DN did not reverse the inhibitory effect of metformin on collagen synthesis, suggesting that the suppressor effect of metformin on collagen production may be independent of AMPK activation [31]. More research is needed to refine our understanding of the molecular mechanisms of antifibrotic activity of metformin.

A central event in cardiac fibrotic remodeling is the accumulation of activated myofibroblasts at the site of injury [37,38,39]. The myofibroblasts, a subset of activated fibroblasts characterized by the expression of α-SMA, are the principal cell type responsible for the synthesis and deposition of collagen during tissue fibrosis [40]. Extensive infiltration of the infarct area with myofibroblasts is a consistent feature of the cardiac remodeling after MI and a prominent characteristic in human myocardial scars [19]. Myofibroblasts produce large amounts of interstitial collagens which alter the geometry of the myocardium and contribute to remodeling in the remote areas of the ventricular wall [18]. According to the results obtained in our study, metformin attenuates mRNA expression of αSMA and collagen type III in cardiac sections from I/R-challenged hearts, suggesting that metformin can counteract the activation of the profibrotic gene program in the failing myocardium. Cardiac fibrosis is closely associated with inflammatory response linked to I/R injury [17]. Complementary to fibrosis analysis, myocardial expression of inflammatory genes was performed to explore the connection between fibrotic remodeling and inflammatory status in postinfarct cardiac tissue. Current work has mainly focused on fibrosis phenotype and expression profile of CD68, a histochemical/cytochemical marker of inflammation associated with the involvement of monocytes/macrophages. Abundant macrophage infiltration into the heart correlates with excessive collagen deposition and ultimately with HF [12]. CD68 is a glycoprotein that is highly expressed in macrophages and other mononuclear phagocytes [13]. We found that metformin reduced the number of CD68-positive cells and CD68-mRNA expression in fibrosis-challenged hearts, suggesting a link between fibrotic and inflammatory responses to I/R injury. Interestingly, we found that in a mouse model of myocardial I/R, metformin treatment for 2 weeks tended to improve cardiac function, but this did not reach significance at 14 days of I/R. We note that we used low doses of metformin (5 mg/kg/day, i.p.) in our study, and perhaps a two-week treatment course is not enough to achieve the improvement of ventricular function. Previously, Xiao et al. reported that chronic treatment for 6 weeks with metformin (200 mg/kg/day, subcutaneously) improves cardiac function in a pressure overload-induced model of HF [31], suggesting that metformin preserves ventricular performance in a time-related manner. Many reports for the different classes of antidiabetics highlight the similar chronic effects of metformin, sodium-glucose cotransporter-2 inhibitors (SGLT-2i) and dipeptidyl peptidase-4 (DPP-4) inhibitors on the heart, which contribute to cardioprotection [41,42]. From a clinical perspective, the detailed investigation of the underlying mechanisms may be an important rational basis for the specific combination of antidiabetic classes.

In summary, metformin counteracts collagen and macrophage accumulation in I/R-challenged myocardium in mice. These findings demonstrate that metformin could protect the heart against abnormal fibrotic and inflammatory remodeling after MI.

## 4. Materials and Methods

### 4.1. Animals

Animal investigations conformed to the Guide for the Care and Use of Laboratory Animals published by the U.S. National Institutes of Health (NIH Publication No. 85-23, revised 1985) and were performed in accordance with the recommendations of the French Accreditation of the Laboratory Animal Care (approved by the local Centre National de la Recherche Scientific ethics committee).

Three-month-old wild-type male C57BI/6J mice purchased from Janvier Labs (Le Genest-Saint-Isle, France) were maintained in a temperature-controlled room (25°C) with a natural day/night cycle and fed a standard chow diet and given ad libitum access to water. Animals were subjected to cardiac ischemia/reperfusion (I/R) for 14 days.

Animals were randomly divided into 4 groups as follows:

(1) Control + PBS (C) group (*n* = 5);

(2) Control + metformin (C + M) group (*n* = 5);

(3) Ischemia/reperfusion + PBS (I/R) group (*n* = 5);

(4) Ischemia/reperfusion + metformin (I/R + M) group (*n* = 5).

Mice then received for 14 consecutive days intraperitoneal injections of vehicle (PBS) or metformin (5 mg/kg/day) in a final volume of 100 μL. Treatment with metformin started after 15 min of reperfusion. The dose–response study was performed to establish a rational choice for metformin concentration in mice.

### 4.2. Experimental Protocol

A mouse model of I/R was used as previously described [43]. The mice were intubated and placed under mechanical ventilation after undergoing general anesthesia with an intraperitoneal injection of ketamine (125 mg/kg) and xylazine (10 mg/kg). A left parasternotomy was performed to expose hearts, and a 0.4 mm polyethylene suture was placed around the left anterior descending coronary artery. A snare was placed on the suture, and regional myocardial ischemia was produced by tightening the snare. After 30 min of ischemia, the occlusive snare was released to initiate reperfusion up to 15 min. Sham-operated control mice underwent the same surgical procedures except that the snare was not tightened.

### 4.3. Echocardiography

Mice were anesthetized with 2% isoflurane and examined with noninvasive echocardiography (Vivid 7 ultrasound, GE; vevo2100 Visual Sonics).

#### Immunofluorescence and Histological Studies

Heart tissues were embedded in optimal cutting temperature compound (OCT) (Sigma-Aldrich, Saint-Quentin-Fallavier, France) under ice-cold 2-methylbutane. For CD68 staining, frozen sections (10 µm) were fixed in 4% paraformaldehyde, followed by permeabilization and blocking in PBS with 0.02% FBS, 1% bovine serum albumin and 0.3% Triton X-100 at RT. Sections were immunostained overnight with anti-CD68 (MCA1957GA) followed by secondary Alexa fluor antibodies (Molecular Probes, Invitrogen, Cergy-Pontoise, France). Images were acquired by confocal Microscope Zeiss LSM 780 and ZEN image analysis software (Zeiss, Marly le roi, France). Four representative assessment zones were established in relation to the infracted area: two peri-infarct zones, one zone in the middle of the infarct and a zone in the posterior segment of the interventricular septum representing a noninfarcted control area of the tissue slice. Quantification of the total number of CD68-positive cells per field of view was performed by counting the total number of positive cells in each field using the ImageJ software. Cardiac fibrosis was assessed on 10 μm heart cryosections using Sirius Red staining aspreviously described [44]. Additionally, fluorescent wheat germ agglutinin (WGA) staining was performed according to [45]. Briefly, cardiac sections were fixed in 4% paraformaldehyde for 10 min and stained with WGA Sigma Aldrich, Saint-Quentin-Fallavier, France solution for 1 h at room temperature in darkness, washed with PBS three times and coverslipped with an antifading mounting medium. The extent of cardiac fibrosis was quantified using ImageJ software (Bio-Rad Services, Marnes la Coquette, France).

### 4.4. Reagents and Antibodies 

Anti-CD68 (MCA1957GA) antibodies from Bio-Rad (Marnes-la-Coquette, France) were used in this study. Oregon Green 488 coupled-wheat germ agglutinin (WGA) labeling and DAPI were from Life Technologies (Villebon sur Yvette, France).

### 4.5. Quantitative RT-PCR Analysis

Total RNAs were isolated from mouse hearts using the GenElute Mammalian Total RNA Miniprep Kit (Sigma Aldrich, Saint-Quentin-Fallavier, France). Total RNAs (500 ng) were reverse transcribed using High Capacity cDNA Reverse Transcription Kit (Applied Biosystems, Villebon sur Yvette, France) in the presence of random hexamers. Real-time quantitative PCR was performed as previously described [46].Theapical regionof the heart was used forRNA extraction. The expression of target mRNA was normalized to GAPDH mRNA expression. Sequences of the forward and reverse primers are as follows:

COL3A1:

Forward—5′-AAGGCGAATTCAAGGCTGAA-3′;

Reverse—5′-TGTGTTTAGTACAGCCATCCTCTAGAA-3′

α-SMA:

Forward—5′-GTCCCAGACATCAGGGAGTAA-3′;

Reverse—5′-TCGGATACTTCAGCGTCAGGA-3′;

CD68:

Forward—5′-AAAGGCCGTTACTCTCCTGC-3′;

Reverse—5′-ACTCGGGCTCTGATGTAGGT-3′;

GAPDH:

Forward—5′-CTTTGTCAAGCTCATTTCCTGG-3′;

Reverse—5′-TCTTGCTCAGTGTCCTTGC-3′;

TNF-α:

Forward—5′-TGGGACAGTGACCTGGACTGT-3′;

Reverse—5′-TTCGGAAAGCCCATTTGAGT-3′;

IL-6:

Forward—5′-GCCCACCAAGAACGATAGTCA-3′;

Reverse—5′-CAAGAAGGCAACTGGATGGAA-3′.

### 4.6. Statistical Analysis

Statistical comparison between groups was performed by one-way ANOVA followed by a Bonferroni’spost hoctest using GraphPad Prism version 5.00 (GraphPad Software, Inc. (San Diego, CA, USA). Data are expressed as mean ± SEM.

## 5. Conclusions

Taken together, these data provide evidence that post-treatment with metformin attenuates cardiac fibrotic remodeling associated with inflammatory responses induced by I/R injury. Moreover, metformin counteracts activation of the gene program that is involved in pathological cardiac remodeling linked to HF.

## Data Availability

https://drive.google.com/drive/folders/1vZJt2gmzRL2CRmBOayNJ8rJzonAmUUmY.

## Supplementary Materials

The following are available online at https://www.mdpi.com/article/10.3390/ijms22179393/s1.
