# Supplementary material for: Metformin Attenuates Postinfarction Myocardial Fibrosis and Inflammation in Mice
Source: Int J Mol Sci. 2021 Aug 30;22(17):9393. doi: 10.3390/ijms22179393 (PMC8430638; doi:10.3390/ijms22179393)
Supplement: Supplementary file 1 [file ijms-22-09393-s001.zip › ijms-1331036-SI.pdf]

**Table S1. Echocardiographic measurements** in mice subjected to 14 days of myocardial I/R. injury.

| **Parameters** | **Control** | **I/R** | **I/R+M** |
|---|---|---|---|
| IVST, d (mm) | 0.9±0.08 | 1.2±0.19 | 1.3±0.09 |
| LVID, d (mm) | 3.9±0.02 | 4.4±0.39 | 4.1±0.04 |
| LVPWT, d (mm) | 0.7±0.01 | 0.8±0.06 | 0.8±0.01 |
| LV vol, d (uL) | 65.8±6.83 | 88.3±19.20 | 73.2±1.62 |
| LV vol, s (uL) | 19.0±1.44 | 57.3±17.81 | 38.3±1.56 |

Data represents the mean ± SEM. IVST, Interventricular septum wall thickness; LVID, left ventricular internal dimension; LVPW, left ventricular posterior wall thickness; LV vol, left ventricle volume; d, diatole; s, systole.
